# Real-time data in cancer registries: Validation of an automated data extraction system

**DOI:** 10.1016/j.isci.2025.113056

**Published:** 2025-07-03

**Authors:** Sylvie A.M. Langhout, Sjoerd J.F. Hermans, Anna J.T. Smit, Elizabeth Berkx, Sophie A. Kurk, Keetje J. Schade, Eduardus F.M. Posthuma, Otto Visser, Jan J. Cornelissen, Peter C. Huijgens, Jurjen Versluis, Maarten van der Wilt, Avinash G. Dinmohamed

**Affiliations:** 1 Performation Healthcare, Zeist, the Netherlands; 2 Erasmus University Medical Center Cancer Institute, Rotterdam, the Netherlands; 3 Department of Research and Development, Netherlands Comprehensive Cancer Organization (IKNL), Utrecht, the Netherlands; 4 Department of Internal Medicine, Reinier de Graaf Hospital, Delft, the Netherlands; 5 Department of Hematology, Leiden University Medical Center, Leiden, the Netherlands; 6 Department of Registration, Netherlands Comprehensive Cancer Organisation (IKNL), Utrecht, the Netherlands; 7 Amsterdam UMC, University of Amsterdam, Department of Hematology, Cancer Center Amsterdam, Amsterdam, the Netherlands; 8 Amsterdam UMC, Vrije Universiteit Amsterdam, Department of Hematology, Cancer Center Amsterdam, Amsterdam, the Netherlands; 9 Erasmus MC, Department of Public Health, University Medical Center Rotterdam, Rotterdam, the Netherlands

**Keywords:** Health information management, Information systems, Data structure

## Abstract

Timely surveillance of cancer treatment requires real-time integration of electronic health records (EHR) data into population-based registries. We validated data from the Datagateway, an automated system that harmonizes structured EHR data across hospitals into a common model to support near real-time enrichment of the Netherlands Cancer Registry (NCR). Data from patients with acute myeloid leukemia, multiple myeloma, lung cancer, and breast cancer were extracted via the Datagateway and compared to NCR data and EHR source data. The system achieved 100% accuracy compared to registered NCR diagnoses, and an accuracy of 95% when comparing new diagnoses to the NCR inclusion criteria. Treatment was correctly identified in all cases, with only 3% of combination therapies misclassified. Laboratory values matched virtually completely; toxicity indicators showed 72%–100% accuracy. Automated real-time EHR data integration using a harmonized model is feasible and reliable, enabling scalable, high-quality support for real-world oncology research.

## Highlights

• Real-time oncology data extracted from EHRs using an automated system
• Common data model harmonizes oncology data from multiple EHR systems
• 100% concordance with NCR diagnoses and 95% accuracy in new diagnosis extraction
• More than 95% accuracy in treatment regimens and laboratory data across cancer types

## Graphical abstract

**Figure:**
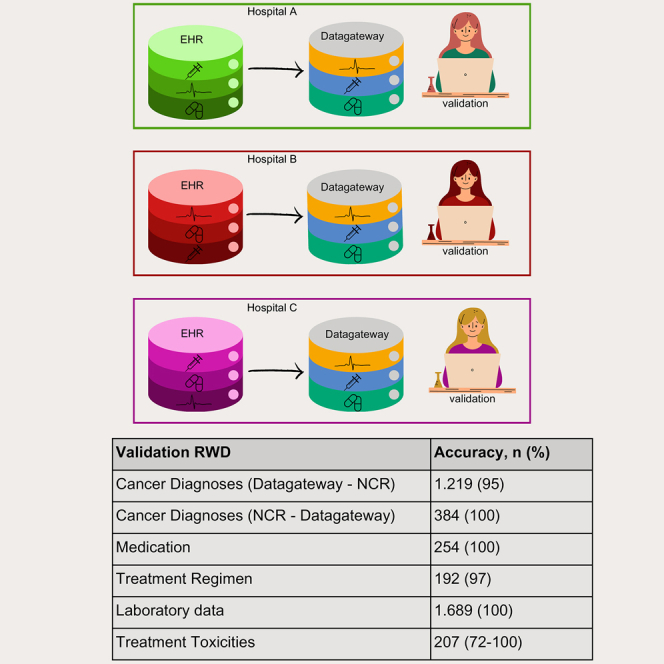

## Introduction

Over the past three decades, population-based cancer registries have become increasingly important.^1^^,^^2^^,^^3^ These registries are a repository of real-world data (RWD) that typically contain data on diagnosis, treatment, and outcome. The data landscape offers insights not only for patients and clinicians but also for researchers, policymakers, insurers, and pharmaceutical industries.^4^^,^^5^ Historically, the emphasis of population-based cancer registries is on epidemiological metrics, including incidence, treatment, survival, and mortality.^6^^,^^7^ However, advances in cancer treatment and cancer prevention challenge these registries to also provide evaluations of diagnostic and therapeutic strategies used in daily practice.^8^^,^^9^^,^^10^ Especially for evaluation of the efficacy and safety of new drugs and strategies, RWD may add to and accelerate the knowledge provided by randomized controlled trials (RCTs).^5^^,^^9^^,^^11^ Furthermore, RWD may be used as supplementary or external control cohorts in the classical clinical trials.^12^ Most population-based registries rely primarily on manual data extraction from electronic health records (EHRs). However, manual registration comes with several limitations, such as being time-consuming and labor-intensive, which will only increase due to the growing demand for the amount and timeliness of data.^13^ Therefore, the development of a data collection method by which registries can automatically collect structured, real-time data from multiple hospitals regarding diagnosis, treatment, and specified outcome measures is needed.^14^^,^^15^^,^^16^

The Netherlands Cancer Registry (NCR) is a population-based registry in which all Dutch cancer patients are manually recorded.^2^^,^^3^^,^^17^ Automated data extraction from EHRs, including disease and treatment characteristics, would enable the NCR to produce near real-time insight into the cancer treatment of Dutch cancer patients. We set out to validate the output of a system that structures EHR data into a common data model, the Datagateway that is able to automatically transfer structured data from EHRs to the NCR.

## Results

### Validation of new diagnoses

#### Prospective validation

A total of 1,287 patient records were evaluated across three hospitals, of which 349 patients were diagnosed with acute myeloid leukemia (AML) and 938 patients with lung cancer. Patient characteristics can be found in Table 1. In total, 1,219 of these patients (95%) met the NCR inclusion criteria. The remaining 68 patients (5%) did not satisfy the NCR criteria for inclusion, primarily due to reasons such as non-Dutch residence (*n* = 11, 16%), a new care trajectory for relapsed or refractory disease (*n* = 27, 40%) or, the patient had a preliminary yet unconfirmed diagnosis which was already recorded as a malignancy in the EHR (*n* = 30, 44%).

**Table 1:** Patient characteristics

Characteristic	All		Acute myeloid leukemia		Lung cancer		Multiple myeloma		Breast cancer	
	Number	%	Number	%	Number	%	Number	%	Number	%
Total patients	1804		517	28,7	1154	64,0	117	6,5	16	0,9

**Sex**										

Females	802	44,5	219	42,4	512	44,4	55	47,0	16	100,0
Males	1001	55,5	298	57,6	641	55,5	62	53,0	0	0,0
Unknown	1	0,1		0	1	0,1		0,0	0	0,0

**Age**										

18–65	570	31,6	211	40,8	285	24,7	62	53,0	12	75,0
65+	1234	68,4	306	59,2	869	75,3	55	47,0	4	25,0

#### Retrospective validation

For the retrospective validation, 384 patient records were evaluated, consisting of 168 AML patients and 216 lung cancer patients. Patient characteristics can be found in Table 1. All patients recorded in the NCR could be retrieved via the Datagateway. More specifically, 342 of these 384 patients (89%) could be identified via the Datagateway with a starting care trajectory and an oncological diagnosis within the same year as was recorded in the NCR. Of the 42 remaining patients (11%), 14 patients (33%) started their care trajectory in the specified hospital in a different year and 28 patients (67%) had a care trajectory registered with a different tumor diagnosis.

### Validation of treatment data

#### Treatment regimens in AML

We validated data on multiple treatment regimens (Table S2) prescribed to AML patients. This validation process involved a total of 254 patients. There was a 100% concordance when comparing the identification of the treatment regimen based on the Datagateway to the previously recorded NCR data or EHR source data.

#### Treatment regimens in MM

In total, 117 patients with multiple myeloma (MM) were included in the validation cohort, and 198 different regimens were validated. Of these 198 regimens, 192 regimens (97%) were correctly identified, and six regimens (3%) were incorrectly identified. The total number of patients per treatment regimen is shown in Tables 2 and S5. Of the six incorrectly identified regimens, two patients were classified as treated with a combination of lenalidomide and dexamethasone (Rd) but had received a higher dose of lenalidomide maintenance (15 mg). Two patients were treated with a regimen not included (lenalidomide—prednisone and melphalan—prednisone) and therefore misclassified as Rd and melphalan, respectively. Furthermore, one patient’s regimen was classified a combination of daratumumab, lenalidomide, and dexamethasone (D-Rd), but the patient was treated with daratumumab (D-mono) and Rd separately. Lastly, one patient was classified as receiving lenalidomide maintenance and D-mono but was treated with lenalidomide maintenance and D-Rd subsequently.

**Table 2:** Performance of EHR extraction of treatment regimen in Multiple myeloma

Treatment regimen	MM patients, n	Accuracy, n (%)
Anti-CD38 based therapy	55	53 (96)
Proteasome inhibitor and IMID-based	22	22 (100)
Proteasome inhibitor-based therapy	16	16 (100)
IMID-based therapy	37	34 (92)
Immunotherapy		
Bispecific antibody therapy	17	17 (100)
CAR-T cell therapy	5	5 (100)
Other	10	9 (90)
Hematopoietic stem cell transplantation	36	36 (100)
Total	198	192 (97)

Anti-CD38 based therapy: daratumumab, bortezomib, lenalidomide, dexamethasone (D-VRd); daratumumab, bortezomib, thalidomide, dexamethasone (D-VTd); daratumumab, bortezomib, cyclophosphamide, dexamethasone (D-VCd); daratumumab, bortezomib, melphalan, prednisone (D-VMP); daratumumab, bortezomib, dexamethasone (D-Vd); daratumumab, lenalidomide, dexamethasone (D-Rd); daratumumab, pomalidomide, dexamethasone (D-Pd); daratumumab monotherapy (D-mono), isatuximab, carfilzomib, lenalidomide, dexamethasone (Isa-KRd); isatuximab, carfilzomib, dexamethasone (Isa-Kd), isatuximab, pomalidomide, dexamethasone (Isa-Pd). Proteasome inhibitor and IMID-based: bortezomib, lenalidomide, dexamethasone (VRd); bortezomib, thalidomide, dexamethasone (VTd); bortezomib, cyclophosphamide, dexamethasone (VCd), pomalidomide, bortezomib, dexamethasone (Pom-Vd), ixazomib, lenalidomide, dexamethasone (IRd). Proteasome inhibitor-based therapy: bortezomib, dexamethasone (Vd), carfilzomib, lenalidomide, dexamethasone (KRd), carfilzomib, dexamethasone (Kd). IMID-based therapy: pomalidomide, cyclophosphamide, dexamethasone (PCd), lenalidomide, dexamethasone (Rd), pomalidomide, dexamethasone (Pd), pomalidomide monotherapy (P-mono), lenalidomide maintenance, iberdomide maintenance. Bispecific antibody therapy: talquetamab, pomalidomdide, daratumumab (Tal-PD), teclistamab (Tec), elranatamab, dexamethasone (Elr), trial medication (Other). Other: elotuzumab, pomalidomide, dexamethasone (Epd), elotuzumab, lenalidomide, dexamethasone (ERd), melphalan, Bridging therapy. Hematopoietic stem cell transplantation: Auto-SCT, Allo-SCT. Abbreviation: MM, multiple myeloma; IMID, immunomodulatory drugs.

#### Laboratory data

We conducted a detailed comparison between laboratory data obtained from two sources: 2,315 items extracted via the Datagateway, and 1,689 items manually collected as part of the phase II TIBET trial (Table S4). Our analysis showed that 0.42% of the items did not match (7 out of 1,689). These discrepancies were due to typographical errors in the manual registration process.

#### Treatment toxicity indicators

The validation of the ten treatment toxicity indicators is shown in Table 3. We observed 100% congruence with actual patient treatment toxicities in three indicators: acute renal failure, ICU admission, and hepatic toxicity. Of the remaining indicators, six indicators demonstrated an accuracy ranging between 50% and 98%. Based on the recorded admission diagnoses, the identification of invasive aspergillosis, invasive candidiasis, and sepsis all had a 100% accuracy. The accuracy of identifying patients with typhlitis and hemorrhage due to treatment was 72% and 92%, respectively. Identification of pulmonary toxicity had a 0% accuracy in both cases. Not all correctly identified patients who were signaled by either the indicator or the registered admission diagnosis could be determined based on both criteria.

**Table 3:** Results of the validation of treatment toxicity indicators in AML

Treatment toxicity (CTCAE)	AML patients (*n* = 254)	Accuracy, *n* (%)
Acute renal failure, n (%)	17 (7)	17 (100)
*Indicator*	*17 (7)*	*17 (100)*
Hemorrhage, n (%)	15 (6)	13 (87)
*Indicator*	*4 (2)*	*2 (50)*
*Admission diagnosis*	*13 (5)*	*12 (92)*
Hepatic toxicity, n (%)	40 (16)	40 (100)
*Indicator*	*40 (16)*	*40 (100)*
ICU admission, n (%)	44 (17)	44 (100)
*Indicator*	*44 (17)*	*44 (100)*
Invasive aspergillosis, n (%)	15 (6)	13 (87)
*Indicator*	*7 (3)*	*5 (71)*
*Admission diagnosis*	*11 (4)*	*11 (100)*
Invasive candidiasis, n (%)	5 (2)	5 (100)
*Indicator*	*4 (2)*	*4 (100)*
*Admission diagnosis*	*3 (1)*	*3 (100)*
Pulmonary toxicity, n (%) (*n* = 91)	8 (9)	0 (0)
*Indicator*	*1 (1)*	*0 (0)*
*Admission diagnosis*	*7 (8)*	*0 (0)*
Sepsis, n (%)	45 (18)	44 (98)
*Indicator*	*43 (17)*	*42 (98)*
*Admission diagnosis*	*10 (4)*	*10 (100)*
Typhlitis, n (%) (*n* = 91)	29 (32)	21 (72)
*Indicator*	*22 (24)*	*19 (86)*
*Admission diagnosis*	*18 (20)*	*13 (72)*
Venous thrombosis, n (%)	11 (4)	10 (91)
*Indicator*	*11 (4)*	*10 (91)*

This table shows the accuracy of the prediction of treatment toxicity, predicted by indicators based on various data items and admission diagnoses recorded. The first column displays the number of patients predicted as a total and as a percentage of the total number of AML patients. The second column shows the accuracy of the prediction as a total and a percentage of the predicted population.

## Discussion

While the initial purpose of the NCR was to support research and policymaking, data usage has partially shifted to other purposes, which results in a need for real-time data. We assessed the feasibility and accuracy of an automated data collection system, the Datagateway, as a system to facilitate automatic EHR data transfer from hospitals to the NCR. We observed that this system was capable of capturing oncological patient data in accordance with the inclusion criteria of the NCR and could also identify corresponding diagnostic data and treatment regimens with high accuracy. However, extracting treatment toxicity demonstrated varying accuracy.

The use of automated data extraction in healthcare research was previously explored and demonstrated high accuracy and efficiency in data collection.^18^^,^^19^ Chauhan et al. conducted an extensive analysis of 427 patients with malignant disease treated with radiotherapy, for whom 25 distinct patient- and treatment-related parameters could be extracted, such as diagnosis code and drug dosage.^18^ Their findings revealed that automated extraction processes were 6,850 times faster compared to manual collection methods. Automated data mining was also applied by Roelofs et al., who demonstrated the feasibility of extracting baseline disease characteristics, such as disease stage.^19^ Similar to these approaches, the Datagateway was able to reliably extract structured data from EHRs, such as diagnosis, treatment regimens, and laboratory results which could considerably reduce the time needed to record these data items in the NCR.

However, combining the structured items to gather complex data gave varied results. Using combinations of drug prescriptions to extract sequential treatment regimens in MM patients proved possible, while combining prescribed drugs and laboratory data to determine treatment toxicity was more difficult. Reasons for this were that drug prescriptions, which were predefined to signal specific disease- and treatment-related infections, were also prescribed for various other indications besides the treatment toxicity of interest. As a result, signaling treatment toxicity for typhlitis, sepsis, invasive aspergillosis, and venous thrombosis had 2%–29% false positives. Admission diagnoses for these indications were more reliable and accuracy ranged between 72% and 100%. However, only treatment toxicities that are severe enough to warrant a hospital admission can be identified with this approach. In addition, as can be derived from Table 3, some patients could only be identified via drug toxicity indicators, and the toxicity events of these patients would be missed if these indicators were not included.

To identify bleeding, pulmonary toxicity, and invasive aspergillosis, we used a keyword matching approach to scan imaging reports for mention of these types of treatment toxicity. However, this approach to identify treatment toxicity had a low accuracy. Alternative methodologies, such as natural language processing (NLP), could improve these methods to obtain information from unstructured data sources. Furthermore, these methods could aid in extracting additional information, such as pathology reports and cytogenetics, which are mainly reported in unstructured text. Research in NLP methodologies, particularly in identifying treatment toxicity indicators, tumor progression, and metastasis, has improved over the past years and strongly suggests that NLP could help notify adverse events from unstructured data.^20^^,^^21^^,^^22^^,^^23^ Ananda-Rajah, Martinez, and colleagues investigated the identification of fungal diseases, including aspergillosis and candidiasis in hemato-oncological patients.^23^^,^^24^ They compared a baseline approach, similar to our approach to identify treatment toxicity based on imaging reports, and greatly improved their sensitivity and specificity using NLP models. Brazeal et al. explored the use of EHR data in an epidemiological study in patients with histologically confirmed advanced adenomatous colorectal polyp. Their research supports the notion that while structured data can be efficiently collected through automated means, differences arise in extracting unstructured data, such as patient history, when comparing manual and automated methods.^25^ Therefore, extensive validation of an NLP model incorporating unstructured data is required to augment the signaling of treatment toxicity indicators in the future.

Since the code used to extract data from EHRs into the data model of the Datagateway is tailored to each specific EHR system (EPIC, Hix, and Nexus) and its versions, and includes customizations for individual hospitals, it is not feasible to provide a single, standardized code within this paper. The system is designed to accommodate variations in EHR structures and processes across different institutions, which means that the code is highly specialized and dynamic. As a result, sharing a common code would not accurately represent the diverse configurations of the various hospital systems involved in this project.

The Datagateway employs the Health Level 7 Fast Health Interoperability Resources (FHIR) and Health Level 7 V3 connections.^26^^,^^27^ The use of FHIR can automate the process and safely transfer data to registries.^28^ Bikkanuri and colleagues found that between 72% and 92% of items in various registries can be automated using FHIR and that the development of more profiles could even result in 100% coverage, leading to faster data acquisition.^13^ These connections facilitate robust data transfer and leverage internationally accepted healthcare terminologies, ensuring interoperability.^28^^,^^29^ Using the FHIR connection, structured data can be seamlessly channeled to registries, such as the NCR, for secure and automated data entry.

In summary, the Datagateway system as a common data model can incorporate real-world EHR data into the NCR in a near real-time and reliable manner. As a result of the automated system, data acquisition is more efficient and more rapid. Gathering unstructured data remains challenging and needs novel technologies and manual validation. Furthermore, registrars will remain essential in validating the data, specifically to assess whether both the structured and unstructured data are concordant and that the complete diagnostic work-up, treatment, and follow-up data of each patient are registered.

### Limitations of this study

This study investigated various aspects, including diagnoses, treatment regimens, laboratory data, and treatment toxicity. However, key information—such as treatment regimens and toxicity details—was not systematically recorded in EHRs. As a result, we had to extract these data through predefined logic, which had implicit limitations when parameters such as laboratory data, diagnostics, and/or drug prescriptions are combined to assign treatment toxicity. In contrast, data that were structurally recorded in the EHRs were more reliable and accurate.

Additionally, the reasons for certain drug prescriptions were not explicitly documented, and drugs were prescribed for indications beyond the treatment toxicity we specifically assessed. To enhance the comprehensiveness of our analysis, expanding the search to include reports and clinical notes using large language models and natural language processing could be beneficial.

Although this study focused on a defined set of data points, there are numerous other areas of interest, such as pathology reports and cytogenetics, and to extract other information such as tumor progression and metastasis. The scope of this study, however, was confined to the selected variables. Future research will extend the investigation to include more unstructured data and incorporate advanced techniques to improve the accuracy of treatment toxicity indicators.

## Resource availability

### Lead contact

Further information and requests for resources and reagents should be directed to and will be fulfilled by the lead contact, Sylvie A.M. Langhout (s.langhout@performation.com).

### Materials availability

This study did not generate any unique reagents.

### Data and code availability

The data reported in this study cannot be deposited in a public repository because in accordance with privacy regulations and institutional policies, the sharing of patient data is forbidden. To request access, contact The Netherlands Comprehensive Cancer Organisation and Performation. Examples of code are made available, any additional information required to reanalyze the data reported in this paper is available from the lead contact(s) upon request.

## Acknowledgments

We extend our sincere gratitude to the dedicated registrars who contributed significantly to the validation of the data in this study, particularly Will van Berkum, Dorette Mekkes, Hedde Rijpstra, Mariëlle Verweij, Jolien Louwerse-Roobol, Ilona Zwinkels, Carolien Peddemors, Della Oskam-Kleinjan, Rolina Harms-Meijering, Eda Bahadir, and Linda Mol. Furthermore, we would like to express our gratitude to the TIBET trial team for the collaboration, especially Dr. M.M.E.M. Bos and Dr. R.M. Bijlsma. Special thanks are due to Corine Korf-van Vliet for her pivotal role in supervising. We are also indebted to Lonneke Vermeulen for her innovative contributions. Research reported in this publication was supported by Oncode Accelerator, a Dutch National Growth Fund project under grant number NGFOP2201.

## Author contributions

P.C.H. and A.G.D. designed the study. O.V. was responsible for the collected data in the Netherlands Cancer Registry. S.A.M.L. performed the main analyses. S.A.M.L. wrote the manuscript with contributions from the remaining authors. S.A.M.L., K.J.S., and S.A.K. developed the queries with clinical input and knowledge from P.C.H., A.G.D., E.F.M.P., and J.V. All authors interpreted the data and read, commented on, and approved the final version of the manuscript.

## Declaration of interests

The authors declare no competing interests.

## STAR★Methods

### Key resources table

**Table:** 

REAGENT or RESOURCE	SOURCE	IDENTIFIER
**Software and algorithms**		

Microsoft SQL Server Management Studio (Version 2019)	Microsoft Corporation (MSFT)	https://learn.microsoft.com/en-us/ssms/download-sql-server-management-studio-ssms
Datagateway	Performation Healthcare Intelligence	This paper

### Experimental model and study participant details

#### Patient cohorts

A total of 1,804 patients were included across four cancer types: acute myeloid leukemia (AML, *n* = 517), lung cancer (*n* = 1,154), multiple myeloma (*n* = 117), and breast cancer (*n* = 16). Overall, 44.5% of patients were female and 55.5% were male; sex was unknown for one patient. Age distribution showed that 31.6% of patients were between 18 and 65 years, while 68.4% were aged 65 or older.

#### Ethics approval and consent to participate

This observational study was conducted following the guidelines set forth by the Central Committee on Research involving Human Subjects (CCMO) in the Netherlands. According to CCMO regulations, this study does not require formal approval from an ethics committee due to its observational nature. This research initiative was made possible by extending pre-existing legal agreements between the Netherlands Comprehensive Cancer Organisation (IKNL), Performation, and the participating hospitals. These extended agreements were meticulously designed to ensure strict adherence to legal and ethical standards, with a paramount focus on safeguarding data privacy and security.

### Method details

#### The Netherlands cancer registry

Nationwide since 1989 and maintained by the Netherlands Comprehensive Cancer Organization (IKNL), the NCR captures over 95% of all newly diagnosed malignancies in the Netherlands.^30^^,^^31^ The NCR derives its notifications of all newly diagnosed malignancies in the Netherlands via the Nationwide Archive of Histopathology and Cytopathology (PALGA), to which all pathological laboratories in the Netherlands report, as well as from the National Registry of Hospital Discharges (i.e., inpatient and outpatient discharges).

The NCR collects patient and disease characteristics such as date of birth, sex, diagnosis, hospital of diagnosis, disease morphology and stage, as well as primary treatment. Trained registrars of the NCR routinely collect these data through retrospective medical records review within 9–12 months post-diagnosis. Approximately 130,000 new patients are recorded annually. Data on the last known vital status (i.e., alive, dead, or, emigration) are updated annually in the NCR via linkage to the Nationwide Population Registries Network. All data entries in the NCR are based on source documents in the patients’ medical records. Standardized procedures for data collection in the NCR align with the guidelines of the International Association of Cancer Registries and European Network of Cancer Registries.

Data collection processes within the NCR comply with Dutch and European Union regulations, including the European General Data Protection Regulation (GDPR).^32^ All Dutch hospitals have given written authorization to IKNL. At diagnosis, patients are informed by their treating physician regarding incorporating their diagnostic and treatment-related data within the NCR. Patients maintain an unequivocal right to withdraw their data from the NCR.

#### The Datagateway

The use of common data models (CDMs) to analyze big datasets, such as using the Observational Medical Outcomes Partnership (OMOP) CDM, is promising.^33^ To extract data from EHR systems, the Datagateway, developed by Performation (Zeist, the Netherlands), serves as a platform adept at harmonizing RWD from the three most frequently used EHR systems in the Netherlands (EPIC, HiX and Nexus) into a CDM.^34^^,^^35^ The data model of the utilized structure of the Datagateway is displayed the Figure S1. Operating within each hospitals’ distinct on-premise server infrastructure, the Datagateway ensures autonomous and secure data management using Microsoft SQL in compliance with individual hospital consents.^36^ Data are extracted from each hospital’s EHR system and transformed into the data model through predefined stored procedures in the SQL database, with hospital-specific configurations to maintain consistency across diverse institutions (Figure S2). Additionally, it employs standardized national and international coding systems (i.e., ICD-10 and LOINC codes), mitigating the differences in EHR database structures.

EHR data can be categorized into structured and unstructured data.^37^ Structured data comprises standardly coded data, such as diagnosis codes and interventions. Many of the structured data elements relate to healthcare administrative tasks, such as hospital admissions, diagnostic procedures, medication prescriptions, and financial claims.^38^ Conversely, unstructured data, which are not recorded into distinct fields by a specific schema, include narrative imaging, pathology reports and healthcare provider’s interpretations in the clinical discourse (e.g., anamnesis). These data from EHRs are inserted into the common data model of the Datagateway, with daily updates of newly added or modified EHR data.

This study adhered to legal and ethical standards by extending existing legal agreements between IKNL, Performation, and the hospitals participating in the study. These extended agreements ensured that all data handling, sharing, and analysis were safe, maintaining data privacy and security standards.

#### Validation process

Validation of the data output was performed in multiple phases to determine if the Datagateway is suited to enable automatic and timely data entry of various items into the NCR. Microsoft SQL queries were developed by data scientists to extract EHR data using the Datagateway. These various clinical data sets comprised patient and disease characteristics, together with treatment information, laboratory test results or, treatment toxicity indicators. After extraction, these items were manually validated by comparing the data extracted via the Datagateway to either similar data elements recorded in the NCR or by comparing the data to the EHR source documents. These validation steps were performed by NCR registrars or hematologists and researchers from the specified hospitals. The various data elements were validated in four different hospitals in the Netherlands, and made available within the on premise servers.

The validation focused on patients diagnosed with acute myeloid leukemia (AML), multiple myeloma (MM), lung cancer and breast cancer. Patients were selected based on disease codes of the International Statistical Classification of Diseases and Related Health Problems 10^th^ Revision (ICD-10), supplemented with diagnosis codes registered in their hospital records. The ICD-10 diagnosis codes and their descriptions are presented in Table S1. Classification of MM was based on ICD-10 classification of plasma cell neoplasms and thus included multiple myeloma, smoldering myeloma, and plasma cell leukemia.

#### Validation of new diagnoses

The initial validation phase assessed the accuracy with which the Datagateway identified newly diagnosed patients. This assessment was divided into prospective and retrospective validations.

##### Prospective validation

During the prospective validation, we assessed whether patients selected by the Datagateway were eligible for inclusion in the NCR. Criteria for inclusion were a new oncological diagnosis coupled with Dutch residency. The registrars of the NCR accessed a list of patient identifiers and their respective diagnoses (i.e., AML or lung cancer) via a secured web application on the hospital’s database server which displayed views from the SQL database. An example of code used to select patients based on ICD-10 diagnosis is available in the Supplementary material (Data S1). The selection contained patients whose initial contact at the current hospital occurred between January 1, 2021, and August 30, 2023. Patients were included four weeks after their initial contact to ensure that all necessary diagnostic activities were completed for the majority of patients. The registrars discerned if the patients selected via the Datagateway were correctly identified and (provisionally) registered in the NCR. Validation of diagnoses was performed between September 2022 to September 2023.

##### Retrospective validation

The retrospective validation aimed to assess the capability of the Datagateway to identify all patients eligible for inclusion in the NCR from EHRs. A list of patients recorded in the NCR, diagnosed with AML between 2018 and 2019, was cross-referenced to a list of patients gathered from EHRs via the Datagateway, whose initial contact was within that same period. The two lists were compared side-by-side and patient identifiers were compared.

#### Validation of treatment data

In addition to diagnostic data, the validation process also encompassed treatment data, including (i) treatment regimens, (ii) laboratory results, and (iii) treatment toxicity indicators. This comparative validation was conducted between EHR data obtained via the Datagateway and existing records in the NCR or EHR source documents data.

##### Treatment regimens in AML

The validation of treatment regimens concerned patients with AML who received a combination of various drugs (Table S2) between January 2021 and September 2023. Validation was performed similarly to the prospective validation of patient diagnosis where treatment details were made available to NCR registrars via a secured web application on the hospital’s server. The selection was made based on relevant ICD-10 diagnosis code and prescription data. Medication was grouped based on their anatomical therapeutic chemical (ATC) codes. The registrars checked if the patients received the treatment for the specified diagnosis and if the treatment dates were correct.

##### Treatment regimens in MM

Similarly, treatment regimens in MM, including triplet and quadruplet regimens, were determined based on prescribed medication. A list of MM treatment regimens is supplied in Table S3. The validation dataset consisted of all treatment regimens of patients with MM whose initial treatment in the selected hospital was between January 2020 and April 2024. Predefined treatment regimens were identified by combining data on drug prescriptions. Treatment was combined when given within the same week. The start and end dates of each regimen were determined based on prescription dates. Validation was performed by comparing the listed regimens for each patient against EHR source data and checking if the listed regimens were correct and that no additional regimens were missing in the Datagateway extracted data.

##### Laboratory results

The laboratory results were validated by obtaining laboratory data from 16 patients diagnosed with ER-positive, HER-2 negative advanced breast cancer, who were included in the phase 2 TIBET trial.^39^ These data included 27 different tests over various treatment intervals for which all laboratory results were collected (Table S4). The data, which comprised basic hematological parameters, blood chemistry, and multiple tumor markers, were manually collected by trained registrars as dictated by the TIBET trial protocol. To ascertain the reliability of the manually gathered data, it was systematically cross-validated against a dataset automatically extracted via the Datagateway. Both datasets were obtained for the specific time intervals during which the patient was included in the trial, ranging from January 2021 to September 30, 2023.

##### Treatment toxicity

Treatment toxicity was determined based on diagnoses registered either as part of a patient’s admission or by deriving toxicity based on various treatment toxicity indicators. Admission diagnoses consist of the registered main diagnosis and other registered relevant secondary diagnoses, as registered based on Dutch hospital registration guidelines.^40^ The various ICD-10 diagnosis codes used to identify treatment toxicity based on the admission diagnosis are displayed in Table S1. To derive treatment toxicity based on indicators we looked at laboratory results and prescribed drugs and used a keyword matching approach to identify treatment toxicity from imaging reports. The definition of the treatment toxicity indicators (Table S6) were defined and evaluated by three hematologists and one academic researcher based on Common Terminology Criteria for Adverse Events (CTCAE).^41^ We looked for treatment toxicity in patients with AML treated with one or a combination of drugs (Table S2) between January 2021 and July 2024. Toxicity was linked to the patient’s treatment if it occurred between the first day of treatment and 30 days after the last day of treatment or until the start of a stem cell transplantation. To validate the results, medical records in the EHR were examined to establish true treatment toxicity at the date signaled via the Datagateway.

### Quantification and statistical analysis

Data extraction and quantification were conducted using structured query language (SQL) in SQL Server Management Studio (SSMS), version 2019. Custom SQL queries were developed to retrieve clinical and demographic data from EHRs.

Aggregate functions were used to compute the number of patients by subgroup (e.g., cancer type, sex, and age group). Following data extraction, percentages were calculated in Microsoft Excel to summarize categorical variables (e.g., distribution of age groups and sex within each cancer type cohort).
