# Supplementary material for: Real-time data in cancer registries: Validation of an automated data extraction system
Source: iScience. 2025 Jul 3;28(8):113056. doi: 10.1016/j.isci.2025.113056 (PMC12303076; doi:10.1016/j.isci.2025.113056)
Supplement: Document S1. Figures S1, S2, Tables S1–S6, and Data S1 [file mmc1.pdf]

iScience, Volume *28*

Supplemental information

Real-time data in cancer registries:

Validation of an automated data extraction system

Sylvie A.M. Langhout, Sjoerd J.F. Hermans, Anna J.T. Smit, Elizabeth Berkx, Sophie A. Kurk, Keetje J. Schade, Eduardus F.M. Posthuma, Otto Visser, Jan J. Cornelissen, Peter C. Huijgens, Jurjen Versluis, Maarten van der Wilt, and Avinash G. Dinmohamed

performation
improving healthcare

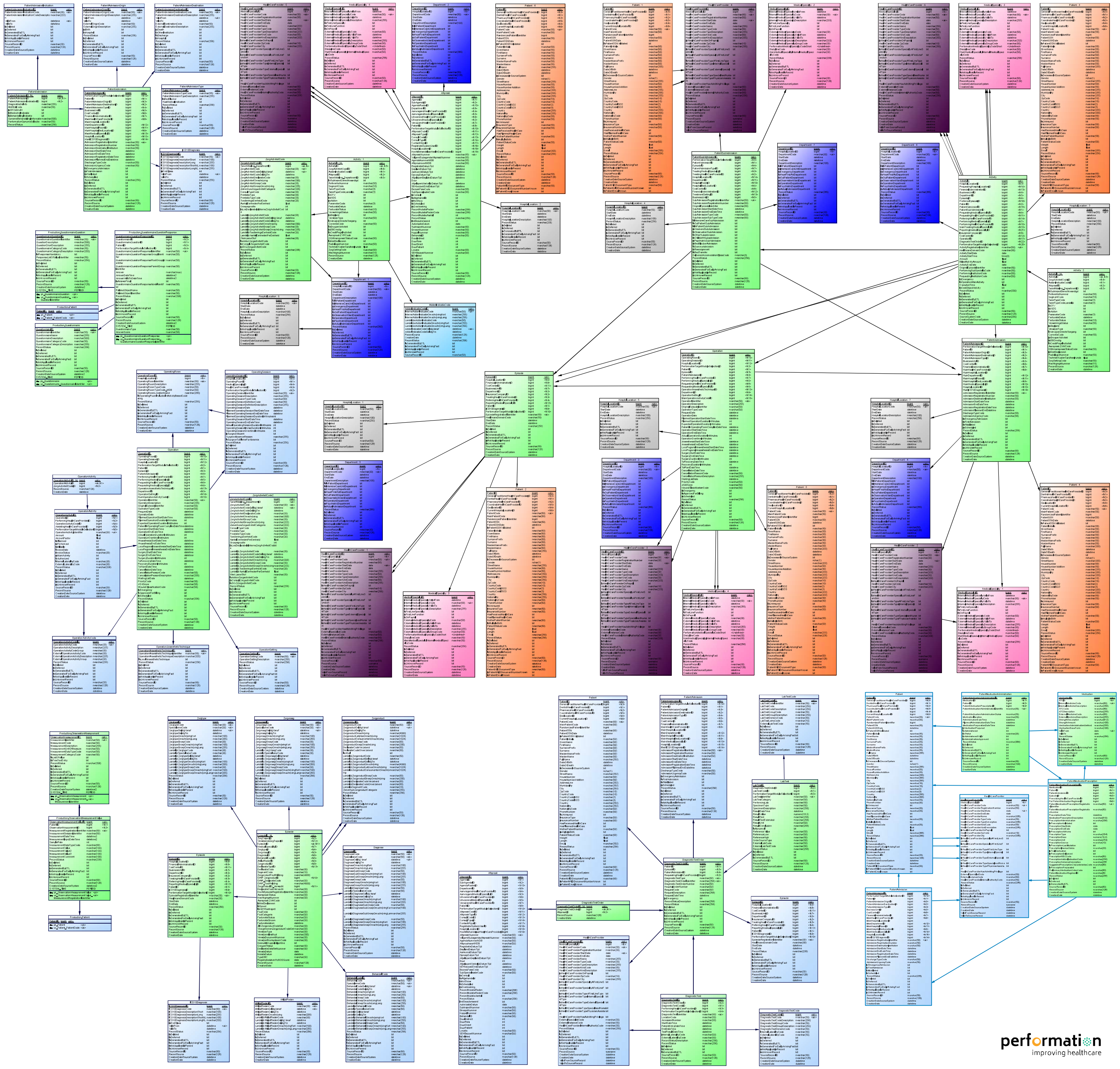

Figure S1: **Data model of the Datagateway.**

Only relevant tables used for this purpose are displayed, however other keys are present in the table column list. Tables relating to the Dutch healthcare system are in Dutch: AddonIndicatieCode = Code used to indicate reason for care which is separately paid for by the insurance, such as for expensive medication; Afspraak = appointment; AfsluitReden = Reason for end of care; Behandelcode = Type of treatment per specialism; Diagnose = Diagnosis; ZorgactiviteitCode = performance descriptions; Zorgproduct = combined care product ; Zorgtype = Type of care, e.g. new, concurrent, IC; Zorgvraag = Reason for the need for care.

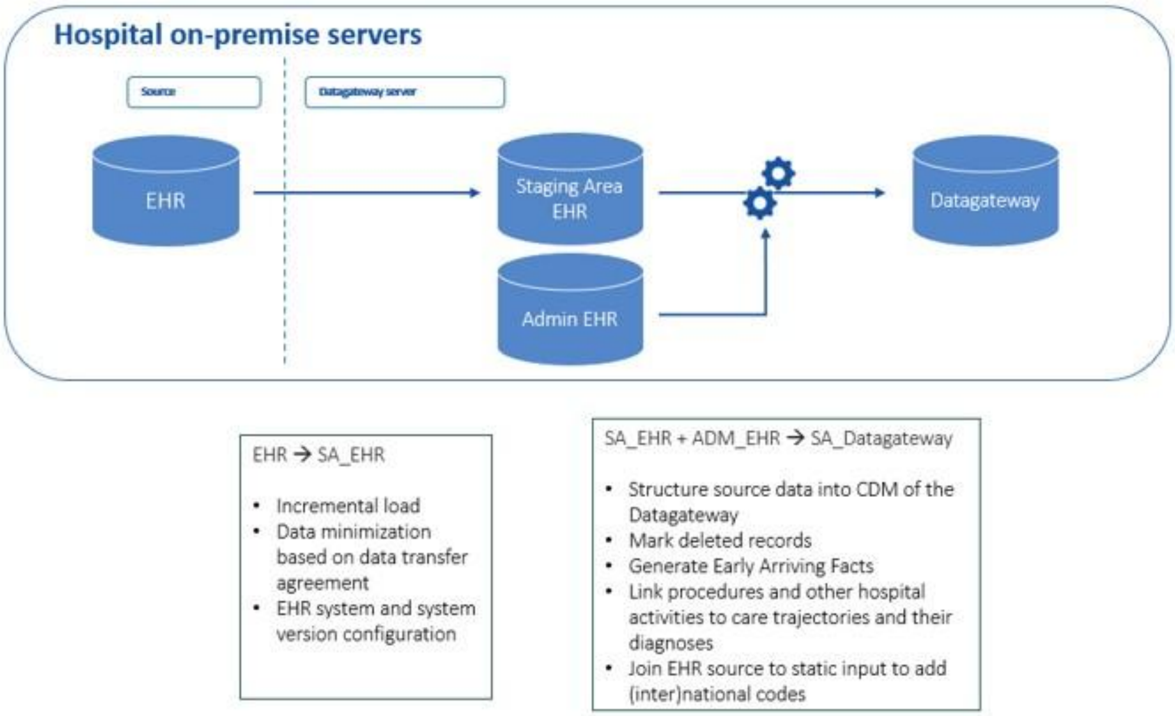

Figure S2: Visualization of the transformation of data from Electronic Health Records into the Datagateway

Data S1: Example of Code to identify patients

```
create table #DiagnosisSelection (
   ICD10DiagnosisCode nvarchar(10)
  , Diagnosis         nvarchar(50)
)
insert into #DiagnosisSelection
(
   ICD10DiagnosisCode
  , Diagnosis
)
Values
  ('C34', 'Lung cancer')
, ('C34.0', 'Lung cancer')
, ('C34.1', 'Lung cancer')
, ('C34.2', 'Lung cancer')
, ('C34.3', 'Lung cancer')
, ('C34.8', 'Lung cancer')
, ('C34.9', 'Lung cancer')
, ('C90.0', 'Multiple myeloma')
, ('C92.0', 'Acute myeloid leukemia')
, ('C92.3', 'Acute myeloid leukemia')
, ('C92.4', 'Acute myeloid leukemia')
, ('C92.5', 'Acute myeloid leukemia')
, ('C92.6', 'Acute myeloid leukemia')
, ('C92.7', 'Acute myeloid leukemia')
, ('C92.8', 'Acute myeloid leukemia')
, ('C92.9', 'Acute myeloid leukemia')
, ('C94.0', 'Acute myeloid leukemia')
, ('C94.2', 'Acute myeloid leukemia')
, ('C94.3', 'Acute myeloid leukemia')
, ('C94.6', 'Acute myeloid leukemia')
, ('D46.2', 'Acute myeloid leukemia')
, ('D46.9', 'Acute myeloid leukemia');

select
   PatientIdentifier =   P.PatientCode
  , DateofBirth =        P.DateOfBirth
  , Initials =           P.PatientInitials
  , Surname =            concat(P.SurnamePrefix, P.Surname)
  , StartCareTrajectory = min(E.StartDate)
  , DiagnosisCode =      concat(ID.ICD10DiagnosisCode, '-', ID.ICD10DiagnosisDescriptionShort)
  , Diagnosis =          DS.Diagnosis
from SA_DataGateway.Production.Episode as E
inner join SA_DataGateway.Production_NL.Episode as E1
   on E1.EpisodeID = E.EpisodeID
inner join SA_DataGateway.Production.ICD10Diagnosis as ID
   on ID.ICD10DiagnosisID = E.ICD10DiagnosisID
inner join #DiagnosisSelection as DS
   on DS.ICD10DiagnosisCode = ID.ICD10DiagnosisCode
inner join SA_DataGateway.Production_NL.Zorgtype as Z
   on Z.ZorgtypeID = E1.ZorgtypeID
inner join SA_DataGateway.Production.Patient as P
   on P.PatientID = E.PatientID
where E.IsDeleted = 0
   and E.IsDeferred = 0
```

```sql
   and Z.LandelijkZorgtypeCode = '11'
group by P.PatientCode
       , P.DateOfBirth
       , P.PatientInitials
       , concat(P.SurnamePrefix,P.Surname)
       , concat(ID.ICD10DiagnosisCode, '-', ID.ICD10DiagnosisDescriptionShort)
       , DS.Diagnosis
order by min(E.StartDate);
```

Table S1: ICD10 codes and descriptions used to identify patients or determine treatment toxicity

ICD10	Description	Diagnosis
A09	Infectious gastroenteritis and colitis, unspecified	Typhlitis
A40	Streptococcal sepsis	Sepsis
A40.0	Sepsis due to streptococcus, group A	Sepsis
A40.1	Sepsis due to streptococcus, group B	Sepsis
A40.2	Sepsis due to streptococcus, group D and Enterococcus	Sepsis
A40.3	Sepsis due to Streptococcus pneumoniae	Sepsis
A40.8	Other streptococcal sepsis	Sepsis
A40.9	Streptococcal sepsis, unspecified	Sepsis
A41	Other sepsis	Sepsis
A41.0	Sepsis due to Staphylococcus aureus	Sepsis
A41.1	Sepsis due to other specified staphylococcus	Sepsis
A41.2	Sepsis due to unspecified staphylococcus	Sepsis
A41.3	Sepsis due to Hemophilus influenzae	Sepsis
A41.4	Sepsis due to anaerobes	Sepsis
A41.5	Sepsis due to other Gram-negative organisms	Sepsis
A41.8	Other specified sepsis	Sepsis
A41.9	Sepsis, unspecified organism	Sepsis
B37.5+	Candida-meningitis	Invasive candidiasis
B37.6+	Candida-endocarditis	Invasive candidiasis
B37.7	Candidal sepsis	Invasive candidiasis/Sepsis
B44	Aspergillosis	Invasive aspergillosis
B44.0	Invasive pulmonary aspergillosis	Invasive aspergillosis
B44.1	Other pulmonary aspergillosis	Invasive aspergillosis
B44.2	Tonsillar aspergillosis	Invasive aspergillosis
B44.7	Disseminated aspergillosis	Invasive aspergillosis
B44.8	Other forms of aspergillosis	Invasive aspergillosis
B44.9	Aspergillosis, unspecified	Invasive aspergillosis
C34	Malignant neoplasm of bronchus and lung	Lung cancer
C34.0	Malignant neoplasm of main bronchus	Lung cancer
C34.1	Malignant neoplasm of upper lobe, bronchus or lung	Lung cancer
C34.2	Malignant neoplasm of middle lobe, bronchus or lung	Lung cancer
C34.3	Malignant neoplasm of lower lobe, bronchus or lung	Lung cancer
C34.8	Malignant neoplasm of overlapping sites of bronchus and lung	Lung cancer
C34.9	Malignant neoplasm of unspecified part of bronchus or lung	Lung cancer
C90.0	Multiple Myeloma	Multiple myeloma
C92.0	Acute myeloblastic leukemia	Acute myeloid leukemia
C92.3	Myeloid sarcoma	Acute myeloid leukemia

C92.4	Acute promyelocytic leukemia	Acute myeloid leukemia
C92.5	Acute myelomonocytic leukemia	Acute myeloid leukemia
C92.6	Acute myeloid leukemia with 11q23-abnormality	Acute myeloid leukemia
C92.7	Other specific forms of myeloid leukemia	Acute myeloid leukemia
C92.8	Acute myeloid leukemia with Multilineage dysplasia	Acute myeloid leukemia
C92.9	Myeloid leukemia, unspecified	Acute myeloid leukemia
C94.0	Acute erythroid leukemia	Acute myeloid leukemia
C94.2	Acute megakaryoblast leukemia	Acute myeloid leukemia
C94.3	Mast cell leukemia	Acute myeloid leukemia
C94.6	Myelodysplastic disease, not elsewhere classified	Acute myeloid leukemia
D46.2	Refractory anemia with excess of blasts [RAEB]	Acute myeloid leukemia
D46.9	Myelodysplastic syndrome, unspecified	Acute myeloid leukemia
H35.6	Retinal hemorrhage	Hemorrhage
I60	Nontraumatic subarachnoid hemorrhage	Hemorrhage
I60.0	Nontraumatic subarachnoid hemorrhage from carotid siphon and bifurcation	Hemorrhage
I60.1	Nontraumatic subarachnoid hemorrhage from middle cerebral artery	Hemorrhage
I60.2	Nontraumatic subarachnoid hemorrhage from anterior communicating artery	Hemorrhage
I60.3	Nontraumatic subarachnoid hemorrhage from posterior communicating artery	Hemorrhage
I60.4	Nontraumatic subarachnoid hemorrhage from basilar artery	Hemorrhage
I60.5	Nontraumatic subarachnoid hemorrhage from vertebral artery	Hemorrhage
I60.6	Nontraumatic subarachnoid hemorrhage from oth intracran art	Hemorrhage
I60.7	Nontraumatic subarachnoid hemorrhage from unsp intracran art	Hemorrhage
I60.8	Other nontraumatic subarachnoid hemorrhage	Hemorrhage
I60.9	Nontraumatic subarachnoid hemorrhage, unspecified	Hemorrhage
I61	Nontraumatic intracerebral hemorrhage	Hemorrhage
I61.0	Nontraumatic intracerebral hemorrhage in hemisphere, subcortical	Hemorrhage
I61.1	Nontraumatic intracerebral hemorrhage in hemisphere, cortical	Hemorrhage
I61.2	Nontraumatic intracerebral hemorrhage in hemisphere, unspecified	Hemorrhage
I61.3	Nontraumatic intracerebral hemorrhage in brain stem	Hemorrhage
I61.4	Nontraumatic intracerebral hemorrhage in cerebellum	Hemorrhage
I61.5	Nontraumatic intracerebral hemorrhage, intraventricular	Hemorrhage
I61.6	Nontraumatic intracerebral hemorrhage, multiple localized	Hemorrhage
I61.8	Other nontraumatic intracerebral hemorrhage	Hemorrhage
I61.9	Nontraumatic intracerebral hemorrhage, unspecified	Hemorrhage
I62	Other and unspecified nontraumatic intracranial hemorrhage	Hemorrhage
I62.0	Nontraumatic subdural hemorrhage	Hemorrhage
I62.1	Nontraumatic extradural hemorrhage	Hemorrhage
I62.9	Nontraumatic intracranial hemorrhage, unspecified	Hemorrhage
J96.0	Acute respiratory failure	Pulmonary Toxicity

J96.00	Acute respiratory failure, unspecified whether with hypoxia or hypercapnia	Pulmonary Toxicity
J96.01	Acute respiratory failure with hypoxia	Pulmonary Toxicity
J96.09	Acute respiratory failure, unspecified	Pulmonary Toxicity
J98	Other respiratory disorders	Pulmonary Toxicity
J98.0	Diseases of bronchus, not elsewhere classified	Pulmonary Toxicity
J98.1	Pulmonary collapse	Pulmonary Toxicity
J98.2	Interstitial emphysema	Pulmonary Toxicity
J98.3	Compensatory emphysema	Pulmonary Toxicity
J98.4	Other disorders of lung	Pulmonary Toxicity
J98.5	Diseases of mediastinum, not elsewhere classified	Pulmonary Toxicity
J98.6	Disorders of diaphragm	Pulmonary Toxicity
J98.7	Respiratory disorders, unspecified	Pulmonary Toxicity
J98.8	Other specified respiratory disorders	Pulmonary Toxicity
J98.9	Respiratory disorder, unspecified	Pulmonary Toxicity
K26.4	Chronic or unspecified duodenal ulcer with hemorrhage	Hemorrhage
K52.1	Toxic gastroenteritis and colitis	Typhlitis
K62.5	Hemorrhage of anus and rectum	Hemorrhage
K92.2	Gastrointestinal hemorrhage, unspecified	Hemorrhage
R04	Hemorrhage from respiratory passages	Hemorrhage
R04.0	Epistaxis	Hemorrhage
R04.1	Hemorrhage from throat	Hemorrhage
R04.2	Hemoptysis	Hemorrhage
R04.8	Hemorrhage from other sites in respiratory passages	Hemorrhage
R04.9	Hemorrhage from respiratory passages, unspecified	Hemorrhage
R57.2	Septic shock	Sepsis
R58	Hemorrhage, not elsewhere classified	Hemorrhage
T81.0	Hemorrhage and hematoma complicating a procedure, not elsewhere classified	Hemorrhage

Table S2: Descriptions of Acute Myeloid Leukemia treatment regimens

ATC Code	Treatment
L01BC07	Azacitidine
L01BB06	Busulfan
L01BB06	Clofarabine
L01AA01	Cyclophosphamide
L01BC01	Cytarabine
L01DB02	Daunorubicin
L01XY01	Daunorubicin combined with cytarabine
L01BC08	Decitabine
L01XX59	Enasidenib
L01CB01	Etoposide
L01BB05	Fludarabine
L01EX13	Gilteritinib
L01DB06	Idarubicin
L01XX62	Ivosidenib
L04AX04	Lenalidomide
L01EX10	Midostaurine
L01DB07	Mitoxantrone
L01XC05	Mylotarg
L01XX66	Selinexor
L01XX52	Venetoclax

Table S3: Multiple Myeloma treatment regimens

Abbreviation	Treatment Regimen
AutoSCT	Autologous stem cell transplantation
AlloSCT	Allogeneic stem cell transplantation
CAR-T (incl. bridging therapy)	CAR-T therapy
D-Kd	Daratumumab, carfilzomib and dexamethasone
D-mono	Daratumumab (with or without dexamethasone)
D-Pd	Daratumumab, pomalidomide and dexamethasone
D-VMP	Daratumumab, Melphalan, prednison and bortezomib
DRd	Daratumumab, lenalidomide and dexamethasone
D-VCd	Daratumumab, bortezomib, cyclophosphamide and dexamethasone
D-Vd	Daratumumab, bortezomib and dexamethasone
D-VRd	Daratumumab, bortezomib, lenalidomide and dexamethasone
D-VTd	Daratumumab, bortezomib, thalidomide and dexamethasone
EPd	Elotuzumab, pomalidomide and dexamethasone
Erd	Elotuzumab, lenalidomide and dexamethasone
Erl	Elranatamab
Ird	Ixazomib, lenalidomide and dexamethasone
Isa-Kd	Isatuximab, carfilzomib and dexamethasone
Isa-KRd	Isatuximab, carfilzomib, lenalidomide and dexamethasone
Isa-Pd	Isatuximab, pomalidomide and dexamethasone
Kd	Carfilzomib and dexamethasone
KRd	Carfilzomib, lenalidomide and dexamethasone
Melphalan	Melphalan
MPT	Melphalan, prednison and thalidomide
MPV	Melphalan, prednison and bortezomib
MPR	Lenalidomide, Melphalan and Prednison
Maintenance Lenalidomide	Lenalidomide (<= 10mg)
Maintenance Iberdomide	Iberdomide
PCd	Pomalidomide, cyclofosfamide and dexamethasone
Pd	Pomalidomide and dexamethasone
Pom-Vd	Pomalidomide, bortezomib and dexamethasone
Rd	Lenalidomide (> 10 mg) and dexamethasone
Tal-PD	Talquetamab, pomalidomide and daratumumab
Tec	Teclistamab
VCd	Bortezomib, cyclophosphamide and dexamethasone
Vd	Bortezomib and dexamethasone
VRD	Bortezomib, lenalidomide and dexamethasone
VTD	Bortezomib, thalidomide and dexamethasone

Table S4: Number of tests and patients per lab test extracted via the Datagateway

Test type	LOINC Code	Test Class	Tests performed, n	Patients, n
ALAT	1742-6	Chemistry	125	16
Albumin	1751-7	Chemistry	80	15
Alkaline Phosphatase	6768-6	Chemistry	125	16
ASAT	1920-8	Chemistry	117	16
Basophils, percentage	30180-4	Hematology	27	15
Basophils, absolute count	26444-0	Hematology	27	15
Bilirubin Total	42719-5	Chemistry	125	16
CA 125	10334-1	Chemistry	6	3
CA 15.3	6875-9	Chemistry	75	15
Calcium	49765-1	Chemistry	88	15
CEA	2039-6	Chemistry	27	8
Eosinophils, percentage	713-8	Hematology	27	15
Eosinophils, absolute count	26449-9	Hematology	27	15
γGT	2324-2	Chemistry	125	16
Glucose	2339-0	Chemistry	94	16
Hemoglobin	718-7	Hematology	153	16
Potassium	6298-4	Chemistry	127	16
Creatinine	38483-4	Chemistry	128	16
LDH	2532-0	Chemistry	125	16
Leucocytes	26464-8	Hematology	148	16
Lymphocytes, percentage	737-7	Hematology	27	15
Lymphocytes, absolute count	26474-7	Hematology	27	15
Monocytes, percentage	744-3	Hematology	27	15
Monocytes, absolute count	743-5	Hematology	27	15
Sodium	2947-0	Chemistry	127	16
Neutrophils, absolute count	26499-4	Hematology	154	16
Platelets	26515-7	Hematology	150	16

ATAL = alanine transaminase, ASAT = aspartate aminotransferase, CA = cancer antigen, CEA = carcinoembryonic antigen, yGT = gamma-glutamyl transferase, LDH = Lactate dehydrogenase

Table S5: Results of MM treatment regiments, for each treatment regimen

Treatment regimen	MM patients, n	Accuracy, n (%)
Anti-CD38 based therapy		
D-VRd	5	5 (100)
D-VTd	2	2 (100)
D-VCd	1	1 (100)
D-VMP	3	3 (100)
D-Vd	3	3 (100)
D-Rd	25	24 (96)
D-Pd	2	2 (100)
D-mono	8	7 (88)
Isa-KRd	4	4 (100)
Isa-Kd	1	1 (100)
Isa-Pd	1	1 (100)
Proteasome inhibitor and IMID-based		
VRd	1	1 (100)
VTd	13	13 (100)
VCd	6	6 (100)
Pom-Vd	1	1 (100)
IRd	1	1 (100)
Proteasome inhibitor-based therapy		
Vd	3	3 (100)
KRd	10	10 (100)
Kd	3	3 (100)
IMID-based therapy		
PCd	7	7 (100)
Rd	13	10 (77)
Pd	4	4 (100)
P-mono	1	1 (100)
Lenalidomide maintenance	10	10 (100)
Iberdomide maintenance	2	2 (100)
Immunotherapy		
Bispecific antibody therapy		
Tal-Pd	1	1 (100)
Tec	9	9 (100)
Erl	5	5 (100)
Other	2	2 (100)
CAR-T cell therapy	5	5 (100)
Other		
EPd	4	4 (100)
ERd	1	1 (100)
Melphalan	1	0 (0)
Bridging therapy	4	4 (100)
Hematopoietic stem cell transplantation		

Auto-SCT	34	34 (100)
Allo-SCT	2	2 (100)
Total	198	192 (97)

Anti-CD38 based therapy: Daratumumab, bortezomib, lenalidomide, dexamethasone (D-VRd); Daratumumab, bortezomib, thalidomide, dexamethasone (D-VTd); Daratumumab, bortezomib, cyclophosphamide, dexamethasone (D-VCd); Daratumumab, bortezomib, melphalan, prednisone (D-VMP); Daratumumab, bortezomib, dexamethasone (D-Vd); Daratumumab, lenalidomide, dexamethasone (D-Rd); Daratumumab, pomalidomide, dexamethasone (D-Pd); Daratumumab monotherapy (D-mono), Isatuximab, carfilzomib, lenalidomide, dexamethasone (Isa-KRd); Isatuximab, carfilzomib, dexamethasone (Isa-Kd), Isatuximab, pomalidomide, dexamethasone (Isa-Pd). Proteasome inhibitor and IMID-based: Bortezomib, lenalidomide, dexamethasone (VRd); Bortezomib, thalidomide, dexamethasone (VTd); Bortezomib, cyclophosphamide, dexamethasone (VCd), Pomalidomide, bortezomib, dexamethasone (Pom-Vd), Ixazomib, lenalidomide, dexamethasone (IRd). Proteasome inhibitor-based therapy: Bortezomib, dexamethasone (Vd), Carfilzomib, lenalidomide, dexamethasone (KRd), Carfilzomib, dexamethasone (Kd). IMID-based therapy: Pomalidomide, cyclophosphamide, dexamethasone (PCd), Lenalidomide, dexamethasone (Rd), Pomalidomide, dexamethasone (Pd), Pomalidomide monotherapy (P-mono), Lenalidomide maintenance, Iberdomide maintenance. Bispecific antibody therapy: Talquetamab, pomalidomdide, daratumumab (Tal-PD), Teclistamab (Tec), Elranatamab, dexamethasone (Elr), trial medication (Other). Other: Elotuzumab, pomalidomide, dexamethasone (Epd), Elotuzumab, lenalidomide, dexamethasone (ERd), Melphalan, Bridging therapy. Hematopoietic stem cell transplantation: Auto-SCT, Allo-SCT. Abbreviation: MM = Multiple Myeloma; IMID = Immunomodulatory drugs.

Table S6: Definitions of treatment toxicity indicators

Indicator	Criteria used to identify the treatment toxicity indicator
Acute renal failure	EGFR rate ≤ 30 ml/min, at least twice within a 72-hour interval and no history of chronic kidney disease
Hemorrhage *	CT or MRI of the brain, colonoscopy or, gastroscopy, with a bleeding reported in the clinical notes
Hepatic toxicity	Increase of ≥5 the upper limit of normal for γGT, ALAT, ASAT or, ALP or ≥3 the upper limit of bilirubin, at least twice within a 72-hour interval
Invasive aspergillosis	CT imaging of thorax and/or bronchoscopy, with aspergillosis reported in clinical notes, or, positive results for aspergillus species from a BAL, followed by antifungal therapy (e.g., azoles)
Invasive candidiasis	Positive blood cultures for candida species followed by antifungal therapy (e.g., echinocandin)
Pulmonary toxicity **	CT imaging of thorax and/or bronchoscopy, with pulmonary toxicity reported, followed by corticosteroid therapy (e.g., prednisone)
Sepsis	Positive blood culture result followed by broad spectrum antibiotics therapy (e.g., piperacillin/tazobactam)
Typhlitis **	CT imaging of the abdomen, followed by a prescription of antibiotics (e.g., metronidazole or piperacilline/tazobactam) and total parenteral nutrition
Venous thrombosis	CT imaging of thorax/abdomen or echo doppler of the legs followed by anticoagulant therapy twice daily (e.g., nadroparine or dalteparine)

* Gastrointestinal and intracranial

** Only in patients treated with Cytarabine

Abbreviations: ALAT = Alanine Aminotransferase, ALP = Alkaline phosphatase, ASAT = Aspartate aminotransferase, BAL = Bronchoalveolar lavage, CT = Computed Tomography, EGFR = Estimated glomerular filtration, MRI = Magnetic Resonance Imaging, yGT = Gamma-glutamyl transferase
